# Automated detection of apoptotic bodies and cells in label-free time-lapse high-throughput video microscopy using deep convolutional neural networks

**DOI:** 10.1093/bioinformatics/btad584

**Published:** 2023-09-29

**Authors:** Kwan-Ling Wu, Melisa Martinez-Paniagua, Kate Reichel, Prashant S Menon, Shravani Deo, Badrinath Roysam, Navin Varadarajan

**Affiliations:** William A. Brookshire Department of Chemical and Biomolecular Engineering, University of Houston, Houston, TX 77204, United States; William A. Brookshire Department of Chemical and Biomolecular Engineering, University of Houston, Houston, TX 77204, United States; William A. Brookshire Department of Chemical and Biomolecular Engineering, University of Houston, Houston, TX 77204, United States; William A. Brookshire Department of Chemical and Biomolecular Engineering, University of Houston, Houston, TX 77204, United States; William A. Brookshire Department of Chemical and Biomolecular Engineering, University of Houston, Houston, TX 77204, United States; Department of Electrical and Computer Engineering, University of Houston, Houston, TX 77204, United States; William A. Brookshire Department of Chemical and Biomolecular Engineering, University of Houston, Houston, TX 77204, United States

## Abstract

**Motivation:**

Reliable label-free methods are needed for detecting and profiling apoptotic events in time-lapse cell–cell interaction assays. Prior studies relied on fluorescent markers of apoptosis, e.g. Annexin-V, that provide an inconsistent and late indication of apoptotic onset for human melanoma cells. Our motivation is to improve the detection of apoptosis by directly detecting apoptotic bodies in a label-free manner.

**Results:**

Our trained ResNet50 network identified nanowells containing apoptotic bodies with 92% accuracy and predicted the onset of apoptosis with an error of one frame (5 min/frame). Our apoptotic body segmentation yielded an IoU accuracy of 75%, allowing associative identification of apoptotic cells. Our method detected apoptosis events, 70% of which were not detected by Annexin-V staining.

**Availability and implementation:**

Open-source code and sample data provided at https://github.com/kwu14victor/ApoBDproject.

## 1 Introduction

In oncology, immunotherapy modalities have demonstrated durable clinical responses and achieved unprecedented success (Schuster *et al.* 2006, Oiseth and Aziz 2017, Esfahani *et al.* 2020). For example, genetically modified lymphocytes provide long-term efficacy against leukemias and lymphomas and earned approval from the US Food and Drug Administration (Le *et al.* 2018, Bouchkouj *et al.* 2019, O’Leary *et al.* 2019). This success has sparked a strong interest in evaluating therapeutics for other types of cancer (including solid tumors) and personalizing treatments (Marofi *et al.* 2021). In this context, the advancement of high-throughput assays is crucial for detailed profiling of cellular activities, especially cell–cell interactions and killing events, for advancing cancer immunotherapy (Ramm 1999, Merouane *et al.* 2015, Lu *et al.* 2019).

In cancer immunotherapy, programmed cell death (PCD) is one of the principal cellular mechanisms for tumor elimination (Lawen 2003, Wong 2011, Panch *et al.* 2019). Therefore, the detection and quantitative profiling of PCD are of central importance for the discovery, validation, and translation of immunotherapeutics. Conventional methods leverage the binding specificity of molecular markers for the robust detection of PCD (Vermes *et al.* 1995, Zhang *et al.* 1997, Kyrylkova *et al.* 2012, Guo *et al.* 2021). For instance, the fluorophore-conjugated Annexin-V marker detects apoptosis by targeting phosphatidylserine (PS) exposure toward the outer leaflet of the apoptotic cell membrane (Lee *et al.* 2013). However, using molecular markers is a restrictive process with multiple drawbacks, including biochemical perturbation of cells, phototoxicity, the need for a dedicated fluorescent channel (a precious resource in high-throughput time-lapse imaging), and signal bleed-through (Niu and Chen 2010, Huh *et al.* 2012, Skylaki *et al.* 2016). In this context, phase-contrast modalities offer essential advantages. They not only avoid the toxicity of fluorescent labels but also provide the potential for earlier detection and a more detailed view of cellular processes. Unfortunately, the complexity, subtlety, and variability of the visual cues in phase-contrast videos have been a barrier to automating the visual analysis. The emergence of deep neural networks makes it possible to develop robust and sophisticated automated computer vision systems for tackling these tasks in high-throughput assays.

**Figure 1.**
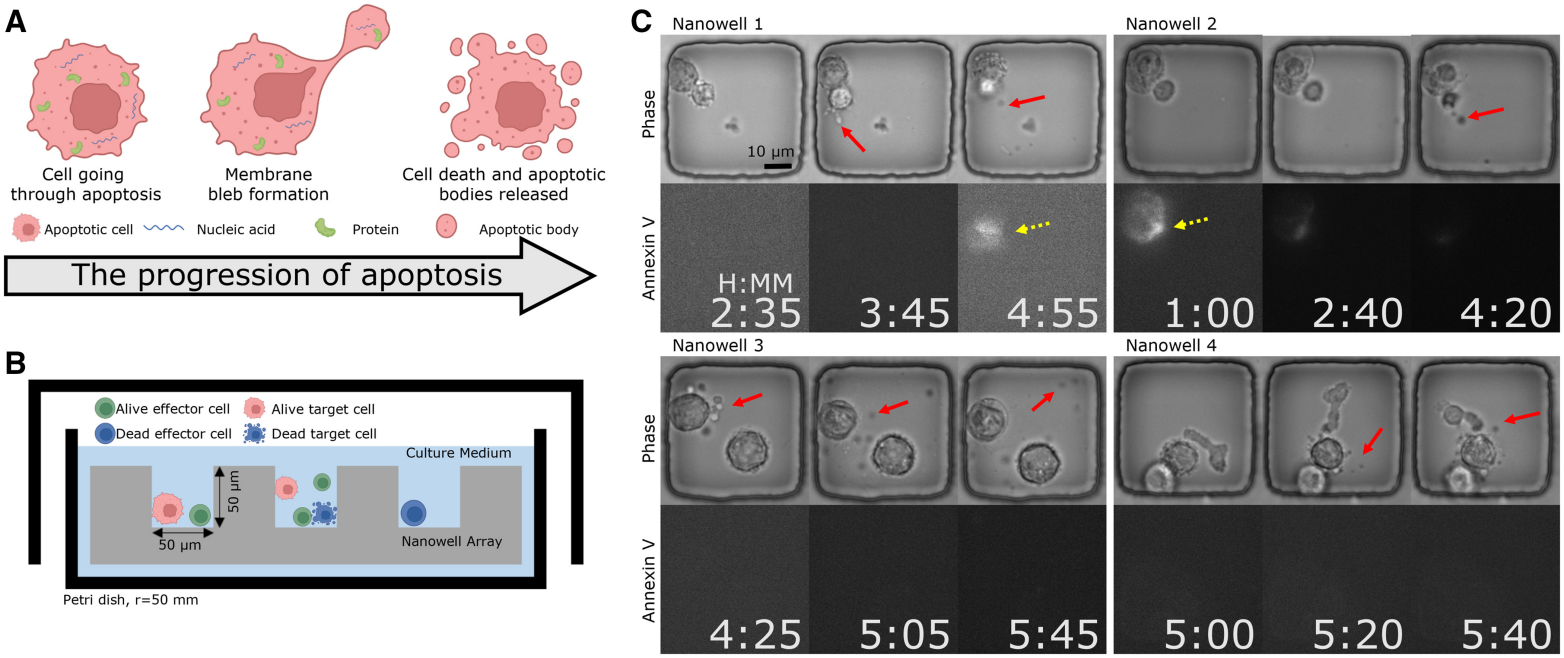
The proposed label-free method for detecting ApoBDs complements apoptosis detection using Annexin-V fluorescence by analyzing the visual indications apparent in phase-contrast imaging (Fig. 7 presents that only 30% of apoptotic cells exhibited a clear Annexin-V signal). Figure created with BioRender.com. (A) ApoBDs are extracellular vesicles produced by apoptotic cells carrying various molecular cargoes of interest. (B) Schematic illustration of the high-throughput TIMING. The system profiles cell–cell interactions at scale. (C) Four sample nanowells illustrate the variable Annexin-V signal within apoptotic cells. In Nanowells 1 and 2, the Annexin-V signal is clear, whereas, in Nanowells 3 and 4, the Annexin-V signal is absent despite the apparent onset of apoptosis in the phase images. The solid arrows highlight ApoBDs release. Dashed arrows highlight cells with Annexin-V staining.

Previous works to detect PCD have explored non-invasive morphology-based apoptosis detection leveraging visual features like nuclei condensation, membrane blebbing, cell shrinkage, and change in cell geometry/texture (Huh *et al.* 2012, Mobiny *et al.* 2020, La Greca *et al.* 2021, Kabir *et al.* 2022). In this study, we focus on the direct detection of membrane-bound vesicles, known as apoptotic bodies (ApoBDs), from apoptotic cell disassembly (Otsuki *et al.* 2003, Xu *et al.* 2019) (Fig. 1A). These small extracellular vesicles (0.5–2.0 μm in diameter) are often visible beyond the cell body, and previous studies on PCD have largely ignored them. Analyzing ApoBDs serves two purposes. First, it can strengthen automated apoptosis detection by leveraging additional visual cues. Second, it can provide valuable data of biological interest. Aside from indicating the induction of apoptosis, ApoBDs also play essential roles in intercellular communication. They carry a variety of molecular cargoes, including nucleotides and proteins (Casciola-Rosen *et al.* 1994, Halicka *et al.* 2000, Schiller *et al.* 2008, Turiák *et al.* 2011), which can induce immunity, tissue regeneration, and even pathogen infection (Kogianni *et al.* 2008, Berda-Haddad *et al.* 2011, Singh *et al.* 2012, Li *et al.* 2014), following phagocytosis. For these reasons, we explored a new approach to ApoBD-based apoptosis detection leveraging deep-learning-based computer vision algorithms.

Convolutional neural networks (CNNs) and vision transformers (ViT) have demonstrated the capability of leveraging abstract features in medical images for high-level tasks like cell phenotyping, disease diagnosis, and lesion detection (Hesamian *et al.* 2019, Lugagne *et al.* 2020, He *et al.* 2022). Previous works on apoptosis detection also proved that deep CNNs sense subtle early changes in the morphologies of apoptotic cells. This work adds a new dimension to the prior studies by analyzing ApoBDs. At first glance, these small and sparsely distributed objects appear as “noise” in images. However, we show that a systematic label-free approach to ApoBD detection and profiling enables the more accurate and sensitive detection of PCD.

The training of deep neural networks on small biomedical datasets relies intensely on human annotation, so we describe resource-efficient methods to train models for tackling distributions of ApoBDs. We created our ApoBD analysis pipeline with sparse labels and deployed it on label-free images. Finally, we provide two examples of how our approach complements conventional apoptosis detection and enables further molecular-level studies to advance cell-based immunotherapy.

## 2 Materials and methods

### 2.1 Image data acquisition and preprocessing

We recorded the interactions between effector [*ex vivo*-expanded tumor-infiltrating lymphocytes (TIL)] and target (Mel526 melanoma cell line) cells within polydimethylsiloxane nanowell arrays fabricated in-house following published protocols (Liadi *et al.* 2015) (Fig. 1B). To differentiate different cell types, we labeled TILs with Green Fluorescent Cell Linker (PKH67) and melanoma cells with Red Fluorescent Cell Linker PKH26 (both from Sigma-Aldrich and at 1 μM) following the manufacturer’s protocol. After loading the cells to the nanowell chips at the concentration of 2 million effector cells and 1 million target cells/ml, we applied a fluorescent apoptosis marker by immersing the entire chip into phenol red-free cell-culture media containing Annexin-V, conjugated to fluorophore Alexa Fluor 647 (from Life Technologies), at a dilution of 1:60. Our imaging system, Time-lapse Imaging Microscopy In Nanowell Grids (TIMING) (Merouane *et al.* 2015, Lu *et al.* 2019), is a high-throughput, multi-channel profiling approach to time-lapse images at the single-cell level. An Axio fluorescent microscope (Carl Zeiss), equipped with 20× 0.8 NA objective and a scientific CMOS camera (Orca Flash 4.0), took images of the chip every 5 min in a humidity/CO_2_ controlled chamber. With ZEN software’s tile function locating the same subset of nanowells in each view during imaging, we obtained time-lapse image sequences of four channels: bright field phase-contrast images; and three fluorescent channels for PKH26, PKH67, and Annexin-V, respectively.

After imaging, we used ZEN software (Carl Zeiss GMBH) to transform the raw images of nanowell subsets into 16-bit Tagged Image File Format, followed by data processing using the TIMING pipeline (Lu *et al.* 2019). The TIMING pipeline consists of deep-learning modules for nanowell detection and cell detection, from which we obtained multi-channel images of individual nanowells. In addition, the TIMING pipeline also provides high-level information on cellular interactions within individual nanowells: first, the cell detection module counts the number of effector and target cells, from which we can find the target cell-containing nanowells. Second, using the Otsu thresholding-based binarization (Otsu 1979), the cropped fluorescent images delineate the immune synapses (ISs). Finally, we define if a cell expresses Annexin-V by computing the intersection over union (IoU) value of the binarized Annexin-V mask and cell mask from the detection module. We defined the IoU of two binary masks, A and B as: $\mathrm{IoU}\left({\mathrm{mask}}_{A},\;{\mathrm{mask}}_{B}\right)=\;\frac{\mathrm{Area}({\mathrm{mask}}_{A}\;\mathrm{and}\;{\mathrm{mask}}_{B})}{\mathrm{Area}({\mathrm{mask}}_{A}\;\mathrm{or}\;{\mathrm{mask}}_{B})}$.

Finally, to validate the quality of our Annexin-V staining as ground truth (GT), we conducted a separate TIMING experiment. The only differences in this experiment are that we used NALM6 tumor cells only and that we imaged the chip in a 3D setup, which led to a lower throughput. We imaged nine different fields of view at 10 different horizontal planes, in one-micron steps, to collect 3D image stacks. Then, we collected image stacks of 84 nanowells where the TIMING pipeline (Lu *et al.* 2019) detected cells. For each Annexin-V image stack, we computed the Pearson correlation coefficient (PCC) of all possible pairs of two slices and took the average over nanowells. As shown in the Supplementary Figure, we observed no PCC lower than 0.95 from the heat map. This confirmed that during high-throughput TIMING imaging, regardless of the choice of the exact 2D image, the Annexin-V signal observed will be a reliable indicator of apoptosis. Since 2D imaging provides reliable Annexin-V label, high imaging rate, and high temporal resolution, it is significantly preferable to 3D imaging.

### 2.2 ApoBD image sequence processing workflow

**Figure 2.**
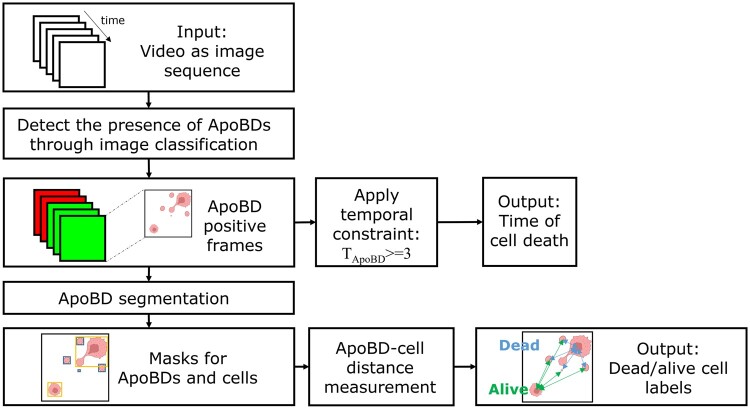
Image analysis workflow for label-free analysis of ApoBDs. Starting from a phase-contrast video image sequence, we first screen nanowells for the presence/absence of ApoBDs using a deep neural network. Next, we look for three consecutive frames containing ApoBDs to quantify the time of cell death. Finally, we segment individual ApoBDs using a second deep neural network and identify the apoptotic cells by association. Figure Ccreated with BioRender.com.

We profiled cell–cell interactions from three Mel526-TIL TIMING experiments with a data processing pipeline consisting of two deep CNN models (Fig. 2). For each image sequence, we first applied an image classifier to detect which frames demonstrate the release of ApoBDs within the nanowells. Next, to find the onset of apoptosis, we used a three-frame temporal constraint to determine if individual frames detected were actual death events or sporadic noise. When we detect ApoBDs in three consecutive frames, we assign the starting frame as the time of onset of apoptosis. Finally, to identify the apoptotic cell within the nanowell, we used the second segmentation model and the TIMING algorithm (Lu *et al.* 2019) to generate instance masks for ApoBD and cells, respectively. After demarcating individual ApoBDs and mapping them to all cells, we assigned the cell with the least average distance to ApoBDs as the apoptotic cell. The output can thus identify the apoptotic cell and the time of induction of PCD.

To map PCD to effector-mediated killing, we performed cell tracking with the TIMING pipeline to determine if there was an effector–target cell contact prior to the detection of ApoBDs and PCD. A qualified contact event must have overlapping masks for more than three consecutive frames. We also examined if the Annexin-V signal lights up for each ApoBD release event by computing the IoU of the cell body mask and the Annexin-V mask generated by Otsu thresholding. We consider the Annexin-V signal with an IoU above 0.1 as valid.

### 2.3 Single-frame classifier for detecting the presence of ApoBD

**Figure 3.**
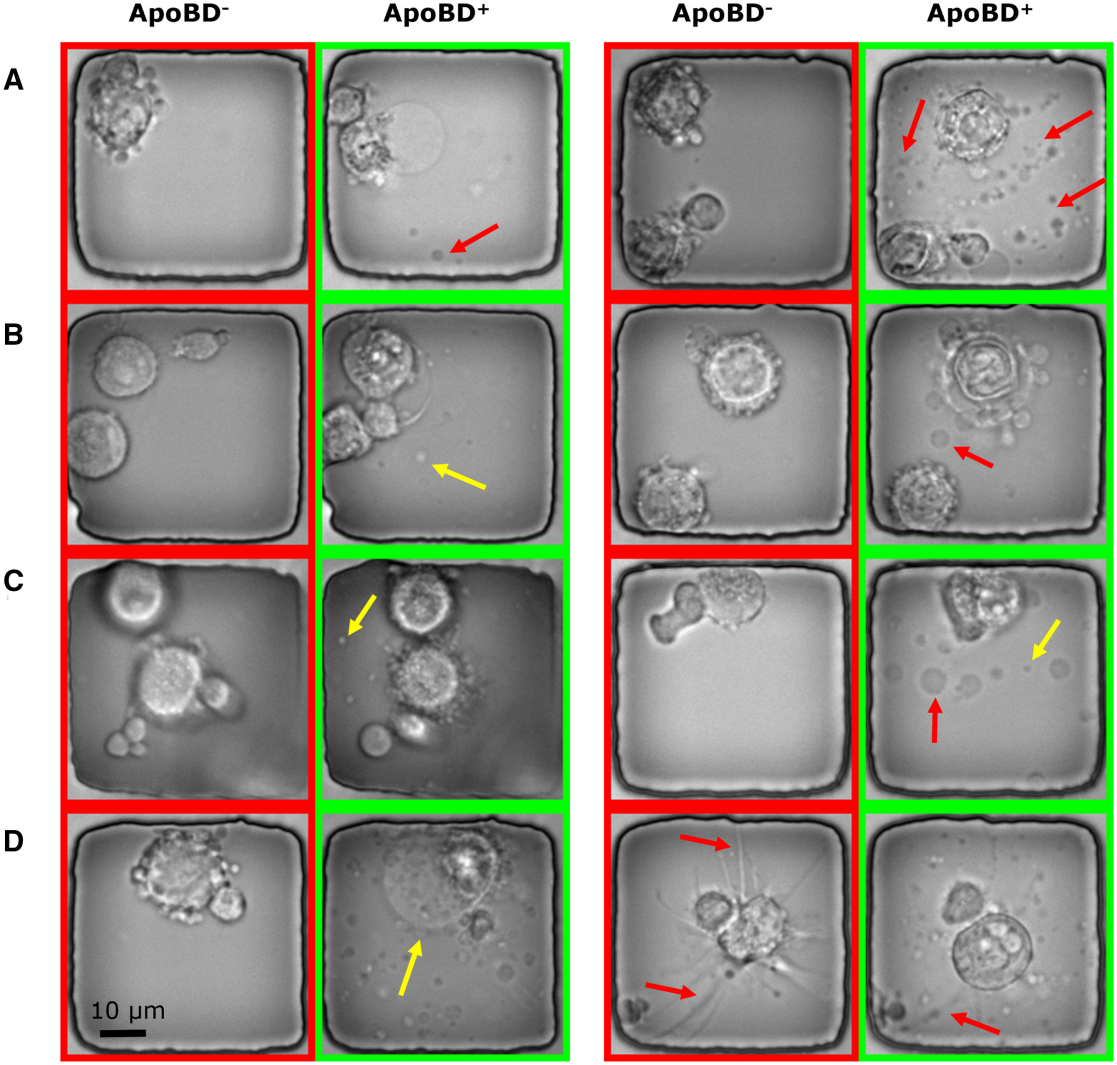
Sample images illustrating the variations in the appearance and complexity of ApoBDs and apoptotic cells in phase-contrast images. Nanowells labeled with ApoBD^-^ do not contain ApoBDs, whereas those labeled with ApoBD^+^ do. (A) An apoptotic cell can release a variable number of ApoBDs (the arrows point out some examples). (B) The ApoBDs are motile and variable in appearance, as exemplified by the ApoBDs indicated by the arrows. (C) Illustrating the variable sizes of ApoBDs. (D) Illustrating the cell-morphological changes that accompany the release of ApoBDs.

Our proposed data processing pipeline starts with an image classifier detecting the presence of ApoBDs. To train such a module, we built the classification dataset for the existence of ApoBD. Interestingly, even though the clear visual cue of ApoBDs was available, we spotted that the Annexin-V staining could not reliably indicate cell death (Fig. 1C), making manual annotation necessary for generating robust GT. Hence, with the help of trained biologists, we obtained a dataset with 7884 images with an ApoBD positive rate of 40%, and we used 75% of these for cross-validation experiments (Fig. 3).

We trained five ImageNet (Krizhevsky *et al.* 2017) pre-trained image classification models to classify the presence of ApoBD: AlexNet, ResNet18, ResNet50 (He *et al.* 2016), Inception (Szegedy *et al.* 2016), and ViT B-16 (Dosovitskiy *et al.* 2020). Previous works (Nawaz *et al.* 2018, Zhang *et al.* 2019, Wang *et al.* 2020, Sarwinda *et al.* 2021, Zhou *et al.* 2022) have demonstrated the capability of these CNNs on medical image classification and made leveraging these off-the-shelf models an efficient choice. We picked AlexNet as a comparison for its lower depth (nine). Two residual networks, ResNet18 and ResNet50, will demonstrate how residual connection increases the number of layers (18 and 50) and benefits network performance. On the other hand, the Inception network extracts high-level features through factorized convolution layers, and the ViT leverages the attention mechanism to learn long-term feature dependency. Taking these two models into consideration enables an analysis of these features. We provide more details about CNN models in Supplementary Figures.

We adopted the original image classification models and set the dimension of the last fully connected layer to two. To compare the models’ performance, we split the dataset to conduct 5-fold cross-validation, during which the images from the same nanowell will stay in the same subset to ensure data independence. The Adam optimizer (Kingma and Ba 2014) with an L2-loss regularization (Phaisangittisagul 2016) coefficient of 10–5 trained all models using the cross-entropy loss function (Mao *et al.* 2023) at a learning rate starting at 10–4 and dropping 5% every 10 iterations. We augmented the dataset with random rotation, brightness, and contrast adjustment operation and trained the model at the batch size of 200 for 50 iterations. To compare different models, we tracked four performance matrices: accuracy, precision, recall, and *F*1-score, against the testing set during cross-validation and took the average of the best performance of each fold. Considering the use of ImageNet for transfer learning, we also normalized the data w.r.t. the mean and variance of the ImageNet dataset.

### 2.4 Determining the onset of apoptosis in image sequences

The onset of apoptosis provides critical information for time-lapse assays. To obtain such temporal information from independent predictions made by the image classifier, we applied a three-frame temporal constraint on classification results. The constraint of three, we chose is an empirical number that fits our data best. To elaborate, for TIMING data, the threshold at one or two frames will be too sensitive and introduce false positive detection due to noise. On the other hand, using a threshold higher than four means the ApoBD has to be recorded for more than 20 min, which can cause false negative results. To test the accuracy of the proposed method, we created synthetic image sequences by concatenating non-repeating images from the validation dataset at random. For each 10-frame long image sequence, we randomly assigned the position and number of ApoBD positive frames. A sequence with more than three consecutive positive frames qualifies as an apoptosis event, with the first positive frame as the time of apoptosis. We created 300 image sequences for apoptosis and non-apoptosis events, and up to half of the frames in a sequence would be ApoBD positive.

Similarly, we applied the same three-frame constraint to the prediction from the trained image classifiers and calculated the difference between apoptosis time prediction and the GT to estimate our approach’s performance. For simplicity, if the prediction misses an apoptosis event or gives a false apoptosis event, we will assign an error of 10 frames (length of sequence).

### 2.5 Mapping ApoBD to the dead cell through segmentation

There are two primary approaches to dead cell identification: Annexin-V marker-based signal intensity thresholding (primarily detecting apoptosis) and morphology-based death detection (broader definition of cell death). Nevertheless, as previously mentioned, the former provides poor robustness in our system, while the latter is limited for three reasons. Firstly, cells releasing ApoBDs can have late or no change in morphology (Supplementary Videos). Secondly, previous morphology-based approaches require the robust GT of Mel-526 cells. However, the unreliable Annexin-V staining makes generating GT difficult. Finally, the ApoBD may introduce noise and negatively affect cell segmentation and, thus, the death detection performance. In other words, an alternate approach is necessary to leverage this critical visual cue for the dead cell. Therefore, we determine dead cells by mapping ApoBDs back to cells, where an ApoBD segmentation model is necessary.

We created a segmentation dataset of ApoBD through the following steps to train an image segmentation model: first, we rescaled the pixel intensity of all images to the range between 0 and 255 (8 bits/pixel) and delineated the cell masks using the TIMING pipeline (Lu *et al.* 2019). The next step was the blob detection using Laplacian of Gaussian kernel to find the centers of each ApoBD. Next, we applied the flood-fill algorithm at each blob center detected to get regions of interest (RoIs) representing individual ApoBDs. To ensure the quality of RoIs, we chose the intensity tolerance that results in the RoI area closest to the circular RoI size from blob detection for each ApoBD. Afterward, we rejected RoIs with low aspect ratio or high area as blob detection can erroneously pick up the edge of nanowell or other noises. The optimal threshold we found for filtering was 0.85 for the aspect ratio and between 20 and 200 pixels for the area. Since the last step may exclude the mask of an ApoBD difficult to segment (false negative), we removed such objects from the image through the median blur using a disk-shaped kernel with a radius of three pixels. We only applied the median kernel within an RoI defined by Grad-CAM algorithms (Selvaraju *et al.* 2017) based on the trained classifier to only remove necessary features. As a result, we created a training dataset with 1816 images and 3572 RoIs for ApoBDs, and a validation dataset with 304 images and 520 ApoBD RoIs.

We trained a MaskRCNN model (He *et al.* 2017) to detect ApoBDs in these processed images, as the model has demonstrated exceptional results in instance segmentation for medical images (Anantharaman *et al.* 2018, Johnson 2020, Jin *et al.* 2021, Padma *et al.* 2022). The model has the ResNet50 architecture for feature extraction backbone, and the weights are pre-trained on ImageNet. After feature extraction, the region proposal network (RPN) (Ren *et al.* 2015) generates region proposals called anchors to differentiate foreground and background, for which we set the Non-Maximum Suppression (Hosang *et al.* 2017) threshold at 0.7 to control the number of overlapping anchors. Within each foreground proposal, the mask head of the network (called MRCNN) generates a pixel-level mask for each object. Hence, the optimization of the MaskRCNN model uses the mask loss of the MRCNN, the bounding box loss of both MRCNN and RPN, and the class loss of both networks as its loss function (He *et al.* 2017). We trained the model for 100 iterations with stochastic gradient descent optimizer (Ruder 2016) with a learning rate of 10–3, learning momentum of 0.9, and L2 regularization coefficient at 10–4. After training, we chose the best set of weights based on the best loss for the validation dataset.

## 3 Results

### 3.1 Deep neural networks accurately detect the presence of ApoBDs in single frames

**Figure 4.**
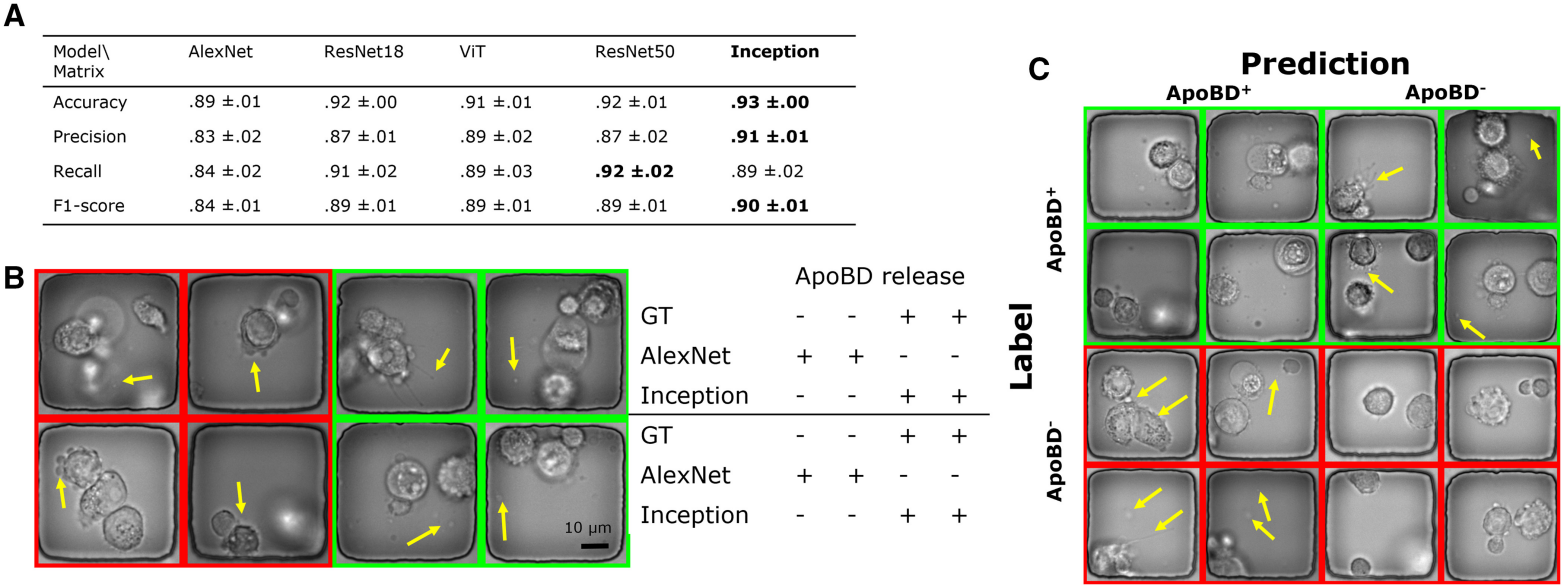
A comparison of the performance of five available deep neural networks. (A) The table showed that the Inception network performed the best in accuracy, precision, and the *F*-1 score. (B) We compared the AlexNet and Inception networks’ performance w.r.t. the GT. Visual examples showed that compared to the AlexNet, the Inception network successfully classified the more complex images with finer details (arrows). (C) Visual examples of Inception networks’ prediction in the format of a confusion matrix showed that despite the overwhelmingly successful performance of the Inception network, there were still rare failures due to details in images (arrows).

To detect the presence of ApoBDs in single frames to screen for PCD, we evaluated the performance of classifiers. We found that the deeper CNNs outperformed other models in the 5-fold cross-validation experiment. The Inception network achieved the best accuracy (93% ± 0%), precision (91% ± 1%), and *F*1 score (90% ± 1%), and the ResNet50 network had the best recall rate at 92% ± 2% (Fig. 4A). Compared with previous reports on biomedical image classification, the performance we achieved using deep CNNs is comparable to those for other datasets (Habibzadeh *et al.* 2018, Pavillon *et al.* 2018, Acevedo *et al.* 2019, Nguyen *et al.* 2019, Ayyappan *et al.* 2020, Mobiny *et al.* 2020). Despite the varying image type and task difficulty, most studies reported an accuracy between 85% and 95%. Consequently, we infer that the CNNs learned meaningful features to detect ApoBDs.

On the other hand, due to the lower depth, AlexNet learned less relevant features and underperformed (Fig. 4A). On the contrary, ResNet18 achieved performance similar to ResNet50, indicating the power of residual connections. Moreover, ViT did not outperform deep CNNs for our task. ViT has outperformed CNNs previously (Bhojanapalli *et al.* 2021, Perera *et al.* 2021, Xiao *et al.* 2023), but such performance requires pre-training with a large dataset, a sizeable training set, and sometimes architecture modification from the original ViT (Krishnan and Krishnan 2021, Zhang and Wen 2021, Tanzi *et al.* 2022). Our results showed that the small dataset size limited ViT’s effectiveness and that the locally distributed features are as relevant as globally distributed ones.

Furthermore, we compared deeper CNN (Inception) and AlexNet by visualizing images that only the former classified correctly (Fig. 4B). We inferred that Inception outperformed in learning abstract, high-level features. Our classification task is difficult as multiple stages of PCD share similar morphologies, and moving ApoBDs are usually in poor focus. For example, in phase-contrast images, circular blobs are visible before (from cell bubbling) and after the release of ApoBD. Therefore, a robust classifier has to learn and detect such abstract concepts. While the AlexNet was likely too sensitive to apoptosis-associated morphologies, the Inception network made more accurate predictions when there were confusing features associated with PCD (left half of Fig. 4B) and out-of-focus ApoBDs (right half of Fig. 4B). In short, deeper CNNs like the Inception network are preferable for our task as they resolve visual ambiguity.

Next, we examined the Inception’s performance with prediction examples in the format of a confusion matrix (Fig. 4C), from which we focused on incorrect predictions to find the source of error. In addition to the aforementioned visual confusion, the lack of temporal information limited our classifiers’ performance. Our trained biological professionals created the dataset with access to the complete time-lapse sequences, but image classifiers took single independent frames as input. However, temporal information strongly affects how humans recognize subtle features in images, especially at the beginning of the ApoBD release. For instance, the information in the subsequent frames can be used to decide if a single out-of-focus blob is a valid ApoBD. In other words, detecting ApoBD in single frames means dividing a time-lapse sequence into multiple independent events and creating noise in the dataset. Such noise leads to task difficulty and a misleading performance matrix affecting both the cell morphology-based and our whole field of view-based approach, which has higher variability. Therefore, considering the source of errors and the nature of the task, we investigated using a temporal constraint to the results from single frames instead.

### 3.2 Temporal constraint and ResNet50 predict the onset of apoptosis

**Figure 5.**
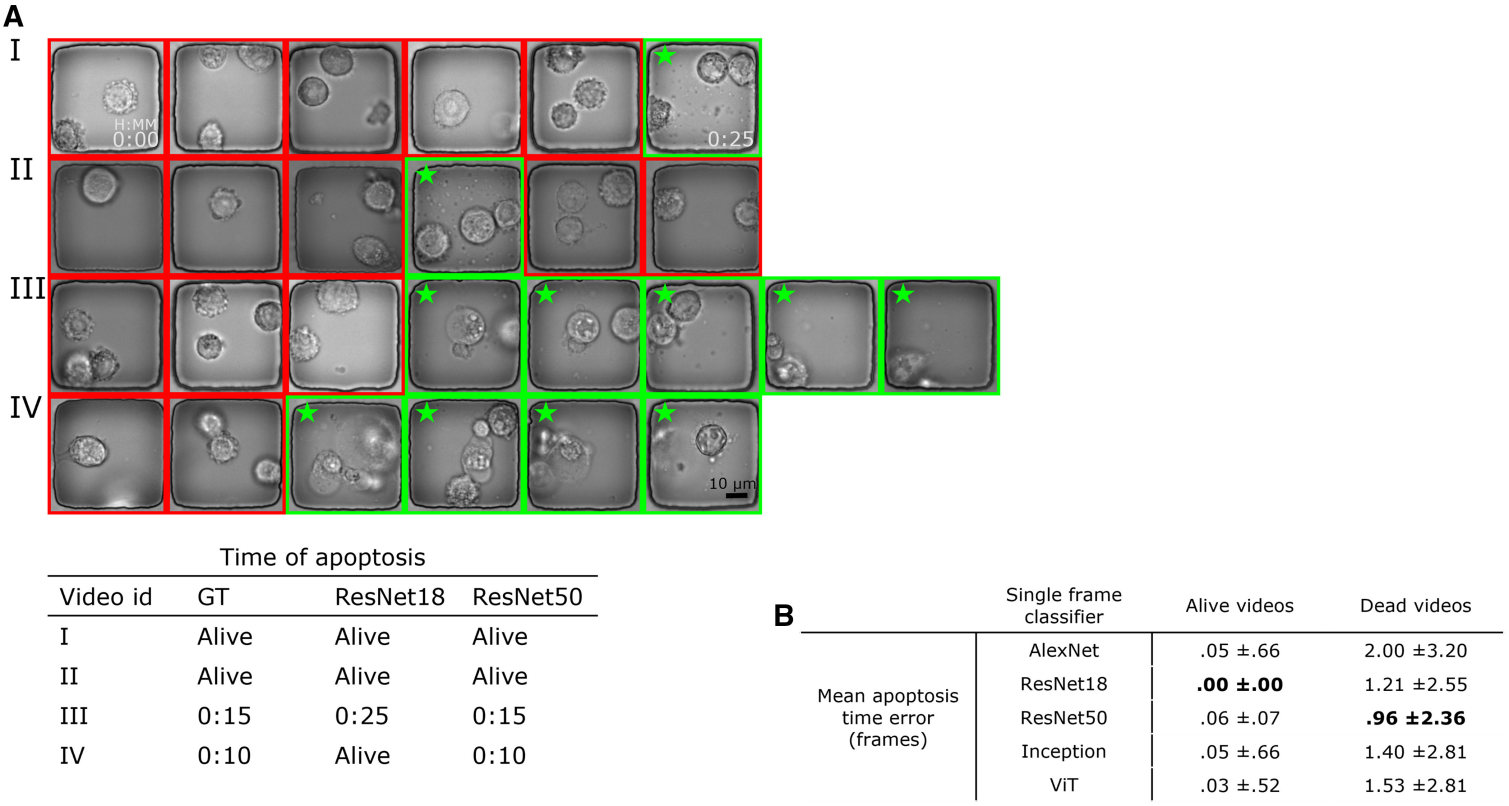
The illustration of our method for assessing the time of cell death by detecting three subsequent frames with ApoBDs presented. In this case, the Resnet50 provided the most effective performance. (A) Visual comparison of the detection performance of Resnet18 and Resnet50. The boxes with star sign highlight nanowells that were labeled to contain ApoBDs. (B) Table of comparative performance of the five neural networks predicting the onset of apoptosis.

After validating the robustness of our classifier in detecting ApoBD, our next step was to determine the accuracy of predicting the onset of PCD using our temporal constraint. The results showed that our approach achieved high accuracy for videos without ApoBD release but may miss a PCD event when the debris is barely visible. For ApoBD-negative events, our approach achieved robust performance with an average error below 0.1 frames with predictions from any classifier (Fig. 5B, the first column). Such performance indicates the robustness of classifiers, and the temporal constraint prevents most false calls of ApoBD release. However, the average error of cell-death events increased to two frames, depending on the classifier selection (Fig. 5B, the second column). As all classifiers performed well on videos without cell death, we chose the model that performed the best on death events, ResNet50, for this study.

ResNet50 reached the most effective detection performance for videos with ApoBD release with the lowest mean error of one frame. Such performance is comparable to predicting apoptosis using only cell morphology in cropped image patch sequence (Mobiny *et al.* 2020). To investigate why ResNet50 outperformed other networks, we inspected the images guided by a biological expert. For example, ResNet18 missed the two bright but out-of-focus ApoBDs in the fifth frame in Video IV in Fig. 5A but ResNet50 correctly classified it as ApoBD positive, thus spotting this apoptosis event accurately. These analyses revealed that ResNet50 could cope with the variations in focus within a time-lapse sequence better than other networks, and ResNet50 gives the best death time prediction for our ApoBD-based pipeline.

### 3.3 We created an ApoBD segmentation model for the efficient identification of apoptotic cells

**Figure 6.**
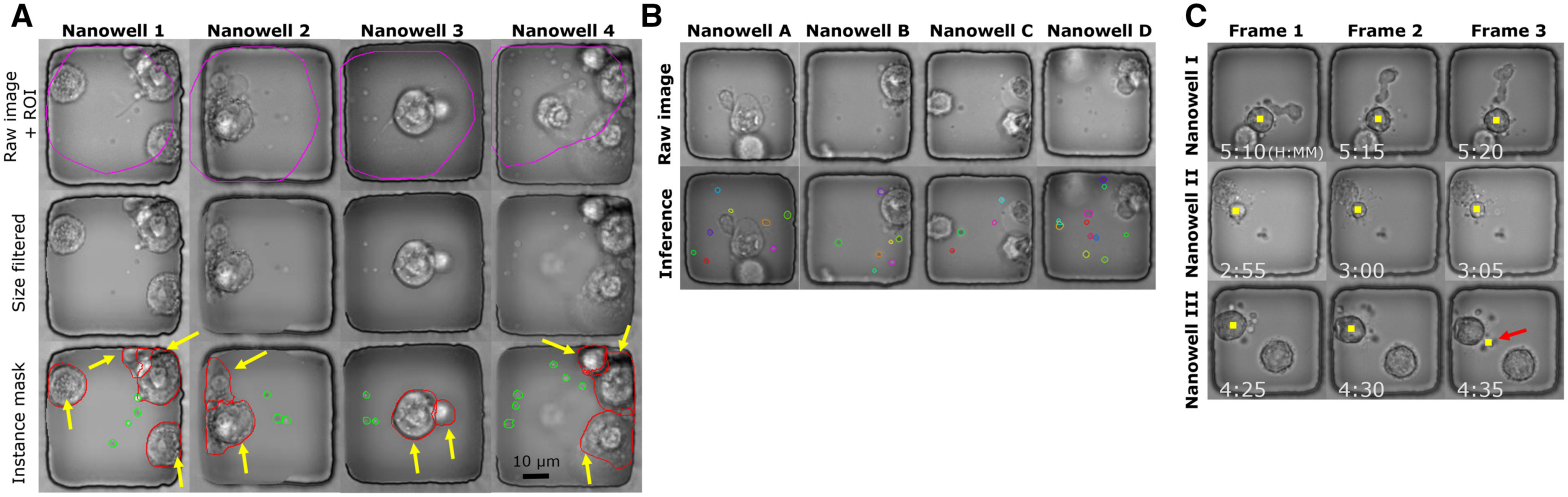
The method for identifying apoptotic cells based on the distribution of ApoBDs. (A) Illustrating the steps for automatically generating segmentations of ApoBDs and cells for four sample nanowells. First, the Resnet50 neural network delineated the RoIs as the contours. The second row shows the result of artifact elimination using size-based filtering of the objects (blobs) in the nanowells. The third row shows the results of instance segmentation with contours with arrow indicating cells and the ones without indicating ApoBDs. (B) Visual examples of effective ApoBD detection using a mask RCNN trained on the segmentations created as in (A), but when run directly on the raw, unfiltered images. (C) The associative process identified apoptotic cells and labeled them with the boxes. We used majority voting across frames to remove rare detection failures highlighted by the arrow.

The final step of our pipeline is to map individual ApoBDs to the dying cell at the onset of apoptosis. To obtain a segmentation model for ApoBDs, we automatically generated a segmentation dataset of ApoBD to train a detection model. As mentioned, Grad-CAM applied on our trained ResNet50 generated the necessary RoI covering most of the ApoBDs and cells (magenta contour in Fig. 6A, Row 1). Furthermore, our success in selective instance removal created filtered images with minimum artifacts (Fig. 6A, Row 2) and proved that the ResNet50 did achieve high classification performance using relevant features. As a result, our approach created a high-quality segmentation dataset in an automated fashion (Fig. 6A, Row 3).

After training, the MaskRCNN model successfully detected ApoBD in phase-contrast images. The model achieved the best average IoU at 0.75 against the testing dataset, which we considered satisfactory for two reasons. First, we created the GT masks automatically and based on pixel intensity, so there will be a difference of several pixels than manual annotation (Gómez-de-Mariscal *et al.* 2019). However, from a practical standpoint, if the predicted centers of ApoBDs are correct, such an error is acceptable. In addition, forcing the network to match our annotation can lead to model over-fitting. Second, detecting small debris in heterogeneous data is more challenging and usually requires post-processing. Hence, considering that our dataset shows high variability in ApoBD morphology (as in Fig. 2), the average IoU we got is more than acceptable for such a difficult task.

As a further validation, we tested MaskRCNN’s robustness by visualizing the inference result for raw, unprocessed images of which the GT was unavailable (Fig. 6B). Based on visual examples, we found MaskRCNN detected ApoBDs regardless of morphology (color and size) and rejected those attached to the cell body (Fig. 6B, nanowells B and C). The segmentation performance was only erroneous for multiple small and overlapping ApoBDs (Fig. 6B, nanowell D). The performance of MaskRCNN indicates that the RoIs we rejected during preprocessing share similar morphology with those we preserved, thus justifying the removal. With the trained MaskRCNN model, we performed segmentation of ApoBDs and measured the distance between debris and cells to identify which cell was going through apoptosis (yellow block in Fig. 6C). The majority vote across three frames helped us reject the incorrect apoptotic cell candidate, which arose from failed cell detection in a single frame (the third frame of Nanowell III in Fig. 6C).

### 3.4 Our approach expands apoptosis detection solely based on Annexin-V

**Figure 7.**
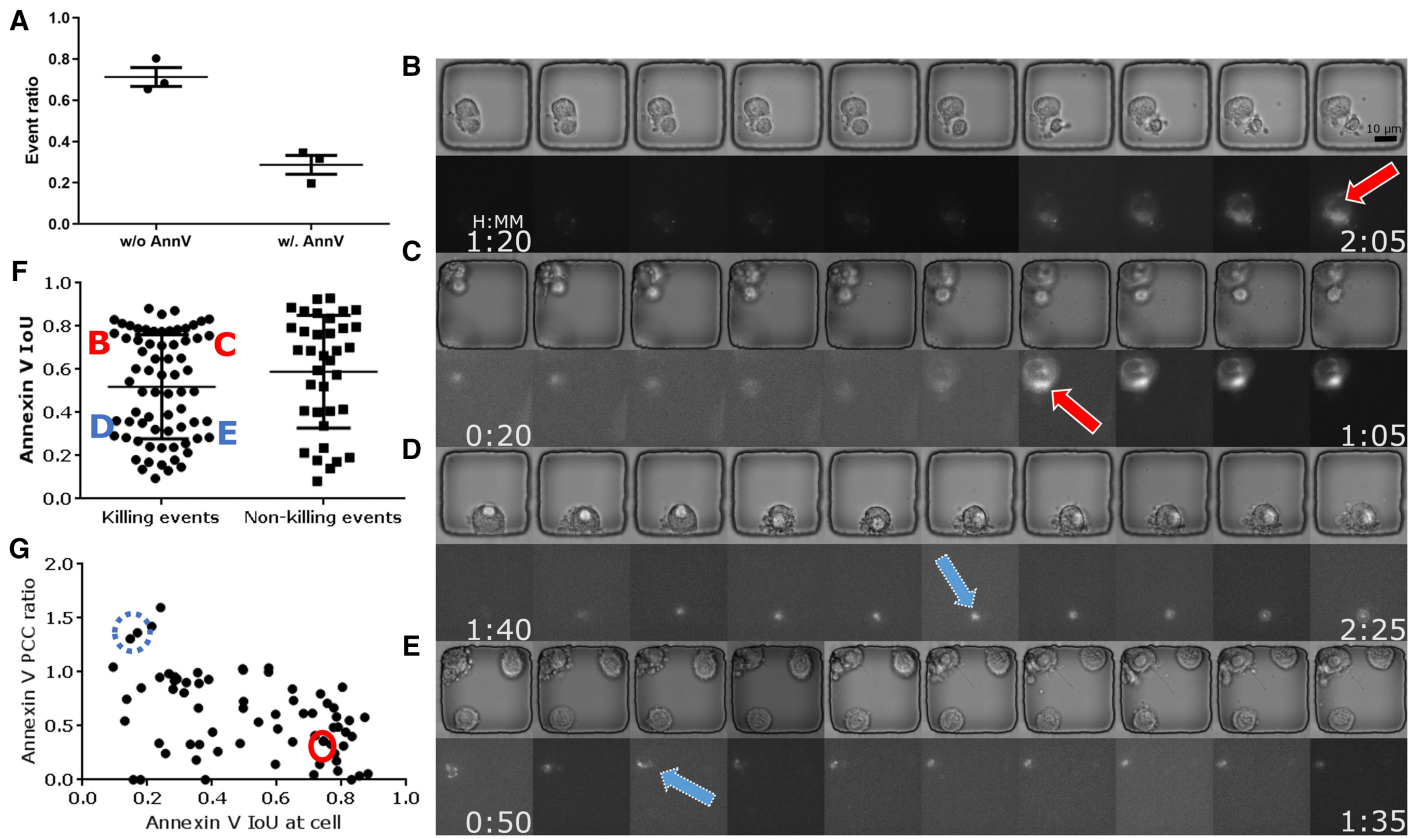
Summary of critical experimental findings. (A) Only about 30% of apoptotic cells we found exhibited a detectable Annexin-V signal, implying that only the proposed ApoBD-distribution-based method can successfully find nearly 70% of them. Therefore, the proposed method offers two significant benefits—the reliable performance of apoptosis detection in a label-free fashion and the ability to handle numerous cases with undetectable Annexin-V signals. (B–E) Four sample cells illustrate Annexin-V signal with different IoU values. Cells D and E have low IoU valueshighlighted by arrows with dashed outline and dashed circle in panel G, while cells B and C display an evenly distributed Annexin-V signal with high IoU indicating the cell body. (F) An IoU-based comparison of the spatial overlap of the Annexin-V signal and the neural-network-based segmentations of apoptotic cells. Signal with low IoU values is harder to detect and *vice versa*. The result showed that more killing events had Annexin-V signal localized than non-killing events. (G) Our analyses showed that the Annexin-V signal polarizing toward the IS has lower IoU during the killing events.

Our pipeline complements the Annexin-V-based apoptosis detection as only about 30% of apoptotic events exhibited a detectable Annexin-V signal. We profiled ApoBD-releasing apoptosis events from three killing assay experiments, and the Annexin-V staining only indicated 32%, 23%, and 28% of events (Fig. 7A). These results suggest that Annexin-V-based staining is insufficient for the reliable detection of PCD, at least with T-cell-based killing. Furthermore, it illustrates the importance of our label-free detection method in detecting 70% of the events. It is impractical to retrieve those missed events using a lower Annexin-V signal threshold, as a threshold lower than 0.1 is too sensitive. Moreover, our approach is independent of cell segmentation, failure of which can lead to wrong measurements for fluorescent signal or cell morphology. In summary, our method demonstrated robustness and achieved superior results compared to conventional methods of identifying PCD.

### 3.5 Annexin-V localized toward the IS in the Mel526-TIL system

Our study of ApoBD-releasing events revealed that the Annexin-V signal localized toward the IS in the Mel526-TIL system. Through visualization, we confirmed that a high IoU (>0.5) of Annexin-V against the cell body indicates an even distribution (Fig. 7C and D), and a low IoU indicates localized staining (Fig. 7E and F). From visual inspection, localized Annexin-V has an affinity to the IS, the interface between effector and target cells in contact. As IS plays a critical role in tumor-killing mechanisms (Jang *et al.* 2015, Xiong *et al.* 2018, Lee *et al.* 2020), we divided all events into two categories: with (killing) or without (non-killing) tumor–effector contact to correlate death marker localization with ISs. Compared with non-killing events, killing events showed a higher frequency of polarized Annexin-V (Fig. 7B). Furthermore, the PCC provides quantified results on Annexin-V localization toward ISs. For events with a broad Annexin-V distribution (IoU>0.6), since ISs are usually of smaller areas than cells unless two cells are tightly conjugated, PCC against most cells exceeds that against the corresponding IS (Fig. 7G). In contrast, for events with a restricted Annexin-V image, PCC against IS surpassed that against the cell, meaning that the bright blob in the fluorescent channel is close to the RoI demarcating IS.

The polarization of the Annexin-V is an interesting phenomenon because it indicates a local increase of lipid density in the cell membrane. Previous work (Rudd-Schmidt *et al.* 2019) showed that the localization of PS toward the presynaptic membrane of a T-cell enhances the protection against perforin, a protein essential for the target cell killing mechanism. To test if localized Annexin-V staining was correlated with the tumor-cell escape from T-cell mediated killing, we collected 17 videos where Annexin-V polarized toward the target cell side of IS. After visual validation, 17 out of 17 events (examples in Supplementary Videos) showed evident change (cell blubbing or ApoBD release) in cell morphology associated with PCD revealing that localized Annexin-V staining at IS was not associated with tumor-cell survival upon T-cell attack.

## 4 Discussion

### 4.1 Toward a robust and comprehensive apoptosis detection

As shown in our results, Annexin-V is inadequate as the sole standard in high-throughput imaging assays as the means of apoptosis detection have increased, and several factors can challenge the robustness of Annexin-V imaging (Lederle *et al.* 2011, Kim *et al.* 2015). First, in addition to apoptosis, the cell membrane also ruptures and exposes PS during necrosis, so Annexin-V staining will cause confusion when classifying subtypes of cell death (Crowley *et al.* 2016). Second, the high background activity and signal multiplexing will affect the image quality of Annexin-V imaging, which is detrimental to high-speed imaging and its downstream analysis. These drawbacks undermine the reliability of Annexin-based assays and the deep-learning models trained on the GT from such assays (Kwak *et al.* 2015). Our results showed that even in our *in vitro* assay, Annexin-V failed as an apoptosis indicator despite apparent visual clues in images. Our ApoBD segmentation model successfully detected apoptosis based solely on time series of phase-contrast images, and the model’s performance in detecting apoptosis is at least as good as standard Annexin-V staining. As shown in Fig. 5, our model utilizes information from multiple frames to accurately determine the time of apoptosis. On the other hand, if time-lapse images are unavailable, the ApoBD model yields an *F*-score of 0.9, which can be further improved using either more training data or integrating information from molecular markers whenever available. By leveraging a combination of molecular markers, widely used cell-death indicators like Calcein AM (Jang *et al.* 2015), and deep-learning powered computer vision algorithms, it is possible to create a multi-modal approach with high performance even for the detection of apoptosis within individual image frames.

### 4.2 Detecting small objects offers data toward a further mechanistic understanding

The performance of our ApoBD segmentation model demonstrated the power of state-of-the-art algorithms but also revealed the value of segmenting small objects in medical images. The segmentation of tiny instances is arduous due to lower resolution, fewer presented features, higher average density, and longer time for human annotation (Hesamian *et al.* 2019, Wang *et al.* 2022). The advancement of computer vision algorithms has enabled the segmentation of small objects, like small brain tumors and exosomes, and the generation of a quantitative heatmap from an image classifier (Selvaraju *et al.* 2017, Ngo *et al.* 2020, Wang *et al.* 2021). With these techniques, imaging assays will achieve better resolution in profiling small objects without relying on fluorescent labels and wasting excessive time. With our detection method, it is possible to conduct a quantitative analysis of the mechanistic role of ApoBD with fewer resources. For example, ApoBDs can carry various types of cargo and induce different biological responses, and a robust segmentation like ours provides high-level information like the engulfment time of ApoBDs. In addition, with no extra label needed for detection, we reduced the effect of signal multiplexing when imaging functional molecules in ApoBD. Our work will help simplify the experimental design to reveal the unknown mechanistic role that ApoBD and apoptosis play.

### 4.3 Conclusion

Including the analysis of ApoBDs in label-free detection of apoptotic cells in high-throughput assays is practical as well as beneficial, and deep neural networks, with their ability to cope with image variability and learning from examples, are enabling. Our multi-scale image analysis strategy using multiple deep networks in a synergistic manner yields more reliable apoptotic cell detection with spatial locations and masks for the ApoBDs that can be used to profile their molecular cargoes. Creating large human-annotated training datasets is a common challenge in deploying deep neural networks. Our study also showed the practicality of generating high-quality training datasets in a resource-efficient manner for training reliable models. Despite the high variability and modest training datasets, the deep CNNs achieved robust performance. Overall, our method can be used in various ways, e.g. complementing Annexin-V-based methods for apoptotic cell detection and profiling ApoBDs to advance imaging assay and immunotherapies.

## Supplementary Material

btad584_Supplementary_Data
